# Low Left-Ventricular Ejection Fraction as a Predictor of Intraprocedural Cardiopulmonary Resuscitation in Patients Undergoing Transcatheter Aortic Valve Implantation

**DOI:** 10.3390/life14040424

**Published:** 2024-03-22

**Authors:** Stephen Gerfer, Clara Großmann, Hannah Gablac, Ahmed Elderia, Hendrik Wienemann, Ihor Krasivskyi, Navid Mader, Samuel Lee, Victor Mauri, Ilija Djordjevic, Matti Adam, Elmar Kuhn, Stephan Baldus, Kaveh Eghbalzadeh, Thorsten Wahlers

**Affiliations:** 1 Department of Cardiothoracic Surgery, Heart Center of the University Hospital of Cologne, Kerpener Straße 62, 50937 Cologne, Germanykaveh.eghbalzadeh@uk-koeln.de (K.E.);; 2 Clinic for Cardiology, Heart Center of the University Hospital of Cologne, Kerpener Straße 62, 50937 Cologne, Germanyvictor.mauri@uk-koeln.de (V.M.);

**Keywords:** TAVR, intraprocedural cardiopulmonary resuscitation (CPR), reduced left-ventricular ejection fraction (LVEF)

## Abstract

Transcatheter aortic valve replacement (TAVR) has become an established alternative to surgical aortic valve replacement (AVR) for patients with moderate-to-high perioperative risk. Periprocedural TAVR complications decrease with growing expertise of implanters. Nevertheless, TAVR can still be accompanied by life-threatening adverse events such as intraprocedural cardiopulmonary resuscitation (CPR). This study analyzed the role of a reduced left-ventricular ejection fraction (LVEF) in intraprocedural complications during TAVR. Perioperative and postoperative outcomes from patients undergoing TAVR in a high-volume center (600 cases per year) were analyzed retrospectively with regard to their left-ventricular ejection fraction. Patients with a reduced left-ventricular ejection fraction (EF ≤ 40%) faced a significantly higher risk of perioperative adverse events. Within this cohort, patients were significantly more often in need of mechanical ventilation (35% vs. 19%). These patients also underwent CPR (17% vs. 5.8%), defibrillation due to ventricular fibrillation (13% vs. 5.4%), and heart–lung circulatory support (6.1% vs. 2.5%) more often. However, these intraprocedural adverse events showed no significant impact on postoperative outcomes regarding in-hospital mortality, stroke, or in-hospital stay. A reduced preprocedural LVEF is a risk factor for intraprocedural adverse events. With respect to this finding, the identified patient cohort should be treated with more caution to prevent intraprocedural incidents.

## 1. Introduction

Transcatheter aortic valve replacement (TAVR) is a well-established alternative to surgical aortic valve replacement (AVR) specifically tailored for higher-risk patients with severe aortic stenosis [1,2]. TAVR has demonstrated favorable postoperative outcomes as well as low mortality rates, and the incidence of stroke and intraprocedural complications has diminished with the increasing procedural volume and expertise of implanters [3,4,5]. This is also why it has also become more common in low-risk patients and demonstrated good results [6]. However, TAVR can be accompanied by life-threatening adverse events such as intraprocedural cardiopulmonary resuscitation (CPR), which may necessitate a surgical bailout, leading to poor outcomes [7]. The occurrence of intraprocedural CPR during the implantation process can be attributed to various factors and has been described as a predictor of in-hospital mortality in emergency TAVR [8,9]. To preemptively address these challenging situations, it is imperative for implanters to know risk factors and thus be prepared for these emergency situations. However, there is a lack of evidence regarding predictors of CPR in the broader TAVR patient population. One potential factor of importance is the left-ventricular ejection fraction (LVEF). We therefore aimed to investigate its role in regard to intraoperative CPR in a diverse all-comer cohort undergoing TAVR procedure at a high-volume center.

## 2. Materials and Methods

### 2.1. Study Population

An analysis of patients in a high-volume center undergoing TAVR was carried out. They were divided into two groups depending on whether their LVEF was above or below 40%: 148 patients presented a lower ejection fraction and 571 patients had an LVEF exceeding 40%. In order to understand potential consequences of the ejection fraction, this study retrospectively analyzed predictors and outcomes of the two groups.

Transfemoral TAVR was generally performed under standby anesthesia, while transapical or transaortic TAVR was performed under general anesthesia. Patients in unstable condition were also put under general anesthesia. Procedures were carried out in a hybrid operation room by the heart team, consisting of a cardiologist and a heart surgeon, accompanied by a cardio-anesthetist.

In the event of hemodynamic instability or CPR, stand-by anesthesia was switched to general anesthesia. CPR was performed based on the suspected cause and resolution of the primary issue. For example, defibrillation or pacemaker implantation was carried out in cases of rhythm disturbances.

In cases of refractory hemodynamic instability and ongoing CPR, extracorporeal mechanical circulatory support was initiated. If necessary, conversion to open heart surgery was performed.

All patients were transferred postoperatively to the intensive care unit (either cardiology or heart surgery) for at least one night and remained there to monitor their postoperative cardio- and pulmonary stability.

### 2.2. Variables of Interest

Preoperative characteristics including demographic data with age and sex, as well as number of comorbidities including coronary artery disease, prior myocardial infarction, peripheral artery disease, arterial hypertension, pulmonary hypertension, chronic obstructive pulmonary disease, diabetes mellitus, hyperlipidemia, atrial fibrillation, prior heart surgery, prior pacemaker and prior stroke were compared between patients with reduced left-ventricular ejection fraction (EF ≤ 40%) and patients with left-ventricular ejection fraction >40%. Furthermore, echocardiography data with mean left-ventricular ejection fraction, aortic diameters, mean and peak pressure gradients were considered relevant parameters between groups.

Intraoperative characteristics with respect to the femoral access route, transapical access, transaortic access, intraoperative mechanical ventilation, operation time, valve-in-valve implantation, rapid pacing, and valvuloplasty and balloon dilation were assessed. Intraoperative cardiopulmonary resuscitation was compared between groups with respect to the onset within the procedure: before rapid pacing, after rapid pacing, before balloon dilation, after balloon dilation, before valve deployment or after valve deployment. Further, intraprocedural defibrillation, heart–lung circulatory support with emergency heart–lung circulatory support, conversion to open heart surgery, pericardial tamponade, valve malpositioning, coronary artery obstruction and ventricular septal perforation were assessed. Postoperative outcomes were analyzed with respect to in-hospital mortality including cardiopulmonary, major bleeding-related death and cerebrovascular death. Furthermore, adverse events such as postoperative sepsis, stroke, transitory ischemic attack (TIA), new onset of atrial fibrillation, new atrioventricular block I°–III° and postoperative new pacemaker implantation due to AV block III° were compared. Additionally, the overall in-hospital and intensive care unit (ICU) stay and red blood cell transfusion, as well as echocardiographic parameter including higher-grade paravalvular leakage, aortic valve endocarditis, aortic valve thrombosis, left-ventricular ejection fraction, aortic valve prostheses mean and peak pressure gradients, were assessed.

### 2.3. Statistical Analysis

The statistical analysis was performed with SPSS Statistics 25 (IBM Corporation, Armonk, NY, USA). As shown in the data tables, all data are given as means and standard deviation (SD) for continuous variables and were analyzed using the Mann–Whitney U test for unpaired data. Normally distributed variables were analyzed using Student’s t-test, whereas the Mann–Whitney U test was used for abnormally distributed variables. Categorical variables are expressed as percentages (cases). Chi-squared or Fisher’s exact tests were used to analyze differences between categorical variables. A *p*-value less than 0.05 was considered to indicate statistical significance. Data are expressed in odds ratio (ORs) and 95% confidence intervals (CIs).

## 3. Results

### 3.1. Preoperative Characteristics of Patients

A total of 719 consecutive patients at a high-volume center undergoing TAVR were analyzed. As all patients underwent preoperative echocardiography, there were no missing data. Preoperative characteristics of patients undergoing TAVR with respect to low left-ventricular ejection fraction (EF ≤ 40%) and controls (EF > 40%) were compared for the following parameters. Age of patients was 80 ± 6.2 years vs. 82 ± 6.1 years, reaching a significant difference (*p* = 0.007) between the low-EF group and the controls. The number of female patients was significantly higher in the control group (*n* = 60 (41%) vs. *n* = 296 (52%), *p* = 0.014). Coronary artery disease (*n* = 102 (70%) vs. *n* = 314 (55%), *p* = 0.002), prior myocardial infarction (*n* = 39 (26%) vs. *n* = 67 (12%), *p* < 0.001) and peripheral artery disease (*n* = 39 (26%) vs. *n* = 82 (14%), *p* < 0.001) occurred significantly more often in the low-EF group. Other preexisting diseases were comparable between groups.

The mean left-ventricular ejection fraction differed between groups (33 ± 7.7% vs. 59 ± 7.8%, *p* < 0.001), as did the aortic annulus diameter (25 ± 2.5 mm vs. 24 ± 2.5 mm, *p* < 0.001), the aortic valve diameter (26 ± 2.3 mm vs. 25 ± 2.1 mm, *p* < 0.001), the aortic valve mean pressure gradient (37 ± 15 mmHg vs. 46 ± 17 mmHg, *p* < 0.001) and the aortic valve peak pressure gradient (62 ± 23 mmHg vs. 74 ± 25 mmHg, *p* < 0.001). Preoperative characteristics are displayed in Table 1.

### 3.2. Intraoperative Characteristics

The intraoperative characteristics are shown in Table 2. Femoral access was chosen more often in the control group (*n* = 116 (78%) vs. *n* = 511 (89%), *p* < 0.001), as well as the transaortic approach (*n* = 6 (4.1%) vs. *n* = 10 (18%), *p* < 0.001). Transapical access was comparable between groups (*n* = 22 (15%) vs. *n* = 54 (9.5%), *p* = 0.057).

The number of patients with the need for a mechanical ventilation was significantly higher in the low-EF group (*n* = 52 (35%) vs. *n* = 108 (19%), *p* < 0.001). The number of patients undergoing valve-in-valve implantation was comparable between groups (*n* = 6 (4.1%) vs. *n* = 23 (4.0%), *p* = 1.000). A rapid pacing for valve implantation was performed more often in the control group with an EF > 40% (*n* = 128 (86%) vs. *n* = 544 (95%), *p* < 0.001) as well as predilation of the native aortic valve with valvuloplasty (*n* = 61 (41%) vs. *n* = 321 (56%), *p* < 0.001).

Intraprocedural cardiopulmonary resuscitation during TAVR occurred more often in the low-EF group (*n* = 25 (17%) vs. *n* = 33 (5.8%), *p* < 0.001). There was no significant difference between the cohorts in the onset of intraprocedural cardiopulmonary resuscitation with respect to timing (Table 2).

The necessity for intraprocedural defibrillation (*n* = 19 (13%) vs. *n* = 31 (5.4%), *p* = 0.002) and heart–lung circulatory support (*n* = 9 (6.1%) vs. *n* = 14 (2.5%), *p* = 0.035) differed significantly. Emergency heart–lung circulatory support (*n* = 1 (0.7%) vs. *n* = 0 (0.0%), *p* = 0.206) and conversion to open heart-surgery (*n* = 3 (2.0%) vs. *n* = 11 (1.9%), *p* = 1.000) was balanced between groups. Other intraprocedural complications did not differ.

### 3.3. Postoperative Outcomes

The postoperative outcomes are displayed in Table 3. The all-cause in-hospital mortality (*n* = 9 (6.1%) vs. *n* = 17 (3.0%), *p* = 0.071) including cardiopulmonary cause (*n* = 1 (0.7%) vs. *n* = 1 (0.2%), *p* = 0.370), major bleeding cause (*n* = 3 (2.0%) vs. *n* = 2 (0.4%), *p* = 0.062) and cerebrovascular cause (*n* = 0 (0.0%) vs. *n* = 2 (0.4%), *p* = 1.000) was comparable between patients with reduced and normal left-ventricular ejection fraction undergoing TAVR.

Postoperative complications with respect to sepsis (*n* = 6 (4.1%) vs. *n* = 14 (2.5%), *p* = 0.272), stroke (*n* = 5 (3.4%) vs. *n* = 10 (1.8%), *p* = 0.208) and transient ischemic attack (*n* = 1 (0.7%) vs. *n* = 5 (0.9%), *p* = 1.000) did not differ between groups.

Postoperative conductance disturbances with a new atrioventricular-block II° (*n* = 6 (4.1%) vs. *n* = 6 (1.1%), *p* = 0.021) occurred more often within the cohort of a reduced left-ventricular ejection fraction. The in-hospital stay (10 ± 7.7 days vs. 8.9 ± 5.4 days, *p* = 0.402) and the intensive care unit stay (4.4 ± 6.5 days vs. 3.3 ± 3.5 days, *p* = 0.298) were comparable between groups. The number of red blood cell transfusions (1.3 ± 2.8 units vs. 0.8 ± 2.4 units, *p* = 0.009) was significantly higher when the ejection fraction was reduced. Postoperative echocardiographic parameters were comparable.

## 4. Discussion

TAVR has emerged as an attractive alternative to a surgical aortic valve replacement (SVR) for older patients and those with comorbidities. However, intraoperative complications such as CPR have been linked to unfavorable outcomes [8]. To avert such complications, it is essential to understand the associated risk factors. The left-ventricular ejection fraction has been shown to have an impact on patient outcome in general heart surgery [10]. We therefore aimed to analyze the role of a reduced ejection fraction in patients undergoing TAVR and their outcome.

Patients with an ejection fraction below 40% exhibited a significantly higher likelihood of undergoing intraoperative CPR (17.0% vs. 5.8%, *p* < 0.001). In addition, they were also more prone to be defibrillated (13.0% vs. 5.4%, *p* = 0.002) and need emergency heart–lung circulatory support (0.7% vs. 0.05%, *p* = 0.206). These findings underscore the heightened vulnerability of patients with a restricted ejection fraction to severe intraoperative complications when compared to these with a higher ejection fraction. This knowledge is crucial for implanters, enabling them to better prepare for potential challenges.

Our study also demonstrated an increased incidence of planned heart–lung circulatory support in patients with a reduced ejection fraction (6.1% vs. 2.5%, *p* = 0.035). Consistent with other research, individuals with a low EF were found to require mechanical support more frequently, such as intra-aortal balloon pump or ECMO [11]. These insights suggest that offering prepared support may be beneficial to ensure a secure valve implantation for these patients. In addition, the rate of emergent heart–lung circulatory support was found to be higher (0.7% vs. 0.0%, *p* = 0.206). This was also found in other studies. Even though the percentage is very low, the mortality rates can be linked to the rates of conversion failure [12]. Therefore, everything should be prepared for emergency support. This also includes a hybrid operation room with fast access to a primed heart–lung machine with a perfusionist on standby and a cardiothoracic anesthesiologist in the operation room for an immediate conversion to open heart surgery and mechanical circulatory support. Emergent cardiac surgery, however, should be avoided at all cost. A meta-analysis found a low rate of emergent surgery, but with a very poor prognosis [13]. Patients with a low ejection fraction are at an even higher risk of a bad outcome and complications and potentially exhibit even higher mortality rates.

Additionally, individuals with an ejection fraction below 40% presented significantly more comorbidities compared to those with a higher EF. These comorbidities included a more frequent history of myocardial infarction (26.0% vs. 12%, *p* < 0.001), a higher incidence of prior heart surgery (31.0% vs. 19.0%, *p* = 0.002) and a higher prevalence of peripheral artery disease (26.0% vs. 14%, *p* < 0.001). Consequently, this may explain the greater tendency to use transapical access (15.0% vs. 9.5%, *p* = 0.057) in these patients, given the limited suitability of femoral artery access routes. Consistently, femoral access for valve implantation occurred significantly less often (78% vs. 89%, *p* ≤ 0.001). Transapical valve implantations have been associated with increased perioperative risks and poorer postoperative outcomes, which could contribute to the elevated rate of intraprocedural CPR [14,15]. This is also why the use of the transapical route has decreased lately and access via the axillary arteria is being used more frequently instead [16,17]. This could be especially advantageous in these high-risk patients, as it is less invasive and faces less risk of myocardial injury [18].

The postoperative outcomes in patients with a lower EF mirror the higher-risk profile of this cohort: they exhibited an increased in-hospital mortality rate (6.1% vs. 3.0%, *p* = 0.071), a greater incidence of stroke (3.4% vs. 1.8%, *p* = 0.208), and longer stays in both intensive care (4.4 ± 6.5 days vs. 3.3 ± 3.5 days) and overall hospitalization (10 ± 7.7 days vs. 8.9 ± 5.4 days). Nevertheless, none of these differences reached statistical significance and all complications were still in a reasonable range. Although it appears that these patients undergo a more complicated periprocedural period as described above, the short-term outcomes do not reflect this complexity and show a reasonable risk of complications.

Moreover, the postoperative LVEF showed a clear increase compared to the preoperative values in both groups. While the rise in the higher-EF group was not significant, with 59 ± 7.8% preoperatively and 60 ± 7% postoperatively, the low-EF group exhibited a substantial improvement in ejection fraction following TAVR, experiencing a noteworthy rise from 33 ± 7.7% to 47 ± 12%. These findings align with existing literature, emphasizing the considerable benefit, especially for low-EF patients, derived from resolving aortic stenosis [19]. Therefore, these patients should be evaluated very carefully and potentially referred to a high-volume center in order to perform a safe procedure and enable an improvement in their ejection fraction.

Furthermore, patients with a low-flow low-gradient aortic stenosis are a special category of TAVR patients. Oikonomou et al. were able to show that even though these patients have a higher one-year mortality, the improvement in LVEF was greater compared to normal-flow aortic stenosis [20]. LVEF recovery was even predicted by a lower baseline LVEF. Therefore, these patients can potentially benefit even more in terms of LVEF.

In addition, while patients with a low EF had a higher risk of a new atrioventricular block II°, the risk of all other kinds of rhythmic disturbances was lower. In addition, patients with a low EF had a slightly lower rate of new pacemaker implantation (11.0% vs. 12.0%, *p* = 0.933). This trend, while not reaching significance, provides encouraging results, considering that ventricular pacing can negatively impact outcomes in individuals with a reduced EF [21].

Furthermore, patients with a reduced ejection fraction exhibited a slightly lower occurrence of higher-grade paravalvular leakage (4.1% vs. 5.8%, *p* = 0.618). This discrepancy contradicts the potentially more challenging intraprocedural situation, highlighting favorable echocardiographic results in patients with a lower ejection fraction.

Moreover, as already mentioned, patients with a reduced EF present a special collective in general. As already described above, they had a more frequent history of myocardial infarction and coronary artery disease (70% vs. 55%, *p* = 0.002). Recent data suggest that CABG surgery outperforms PCI in patients with multivessel coronary artery disease and an ischemic left-ventricular dysfunction [22]. Therefore, these patients should also be evaluated regarding combined CABG and SAVR surgery when an intervention of their coronary artery disease is needed.

In addition, these patients more often require mechanical support in the form of assistance devices. In general, patients with reduced ejection can be evaluated for long-term assistance devices. However, they also often need short-term help, for example, when they decompensate acutely. As already described above, intraoperative emergent as well as planned heart–lung support was more often needed in the patients with a low EF. No other assistance device was used in our study. However, patients might benefit from planned support with an Impella as it has has been described in other cardiac surgeries [23]. Therefore, preoperative evaluation should be carried out.

This collective evidence shows the dynamics of postoperative outcomes in relation to different EF levels, emphasizing the importance of comprehensive patient assessment and tailored interventions. Patients with a lower LVEF represent a higher-risk collective undergoing TAVR and should thus be treated as such. The awareness of complications like intraprocedural CPR or conversion to open heart surgery should be heightened.

### Study Limitations

While valuable in its insights, this study does have certain limitations. Firstly, it was structured as a retrospective, nonrandomized investigation. Moreover, it solely relied on data from a single center, resulting in a relatively small patient cohort. Additionally, our focus was primarily on in-hospital short-term outcomes, and thus comprehensive long-term survival and patient follow-up data are lacking. Therefore, the presented data have to be taken with caution, as the study design was not powered for outcome measurements.

## 5. Conclusions

Patients with a reduced ejection fraction face an elevated risk of intraprocedural CPR during TAVR compared to patients with an ejection fraction above 40%. Postoperative outcomes reveal an increased mortality and a greater incidence of strokes within the reduced-LVEF cohort, indicative of a more demanding perioperative phase. Therefore, it is essential for implanters to approach these patients with heightened caution.

Despite the outcome, however, these patients demonstrate substantial benefit from TAVR, with a significant improvement in their ejection fraction. Therefore, they should be evaluated early on for suitability and may be transferred to higher-volume centers with more experience in order to minimize postoperative complications. Further comprehensive, long-term studies are necessary to confirm these results.

## Figures and Tables

**Table 1:** Preoperative characteristics.

Preoperative Characteristics	EF ≤ 40% (*n* = 148)	EF > 40% (*n* = 571)	*p*-Value
Age, years	80 ± 6.2	82 ± 6.1	0.007
Female, *n* (%)	60 (41)	296 (52)	0.014
Coronary artery disease, *n* (%)	102 (70)	314 (55)	0.002
Prior myocardial infarction, *n* (%)	39 (26)	67 (12)	<0.001
Peripheral artery disease, *n* (%)	39 (26)	82 (14)	<0.001
Arterial hypertension, *n* (%)	141 (95)	536 (94)	0.518
Pulmonary hypertension, *n* (%)	101 (68)	347 (61)	0.095
Chronic obstructive pulmonary disease, *n* (%)	48 (32)	153 (27)	0.173
Diabetes mellitus, *n* (%)	59 (40)	178 (31)	0.045
Hyperlipidemia, *n* (%)	96 (65)	379 (66)	0.730
Atrial fibrillation, *n* (%)	83 (56)	251 (44)	0.008
Prior heart surgery, *n* (%)	46 (31)	107 (19)	0.002
Prior pacemaker, *n* (%)	35 (24)	67 (12)	<0.001
Prior stroke, *n* (%)	20 (14)	85 (15)	0.673
Left-ventricular ejection fraction, %	33 ± 7.7	59 ± 7.8	<0.001
Aortic annulus diameter, mm	25 ± 2.5	24 ± 2.5	<0.001
Aortic valve diameter, mm	26 ± 2.3	25 ± 2.1	<0.001
Mean pressure gradient, mmHg	37 ± 15	46 ± 17	<0.001
Peak pressure gradient, mmHg	62 ± 23	74 ± 25	<0.001
Systolic blood pressure, mmHg	118 ± 13	120 ± 16	0.064
Diastolic blood pressure, mmHg	56 ± 8.3	57 ± 8.9	0.639

Data are expressed as mean ± standard deviation (SD), median (range) or percentage (count) as indicated.

**Table 2:** Intraoperative characteristics.

Intraoperative Characteristics	EF ≤ 40% (*n* = 148)	EF > 40% (*n* = 571)	*p*-Value
Femoral access, *n* (%)	116 (78)	511 (89)	<0.001
Transapical access, *n* (%)	22 (15)	54 (9.5)	0.057
Transaortic access, *n* (%)	6 (4.1)	10 (18)	<0.001
Mechanical ventilation, *n* (%)	52 (35)	108 (19)	<0.001
Operation time, minutes	93 ± 39	88 ± 33	0.257
Valve-in-valve implantation, *n* (%)	6 (4.1)	23 (4.0)	1.000
Rapid pacing, *n* (%)	128 (86)	544 (95)	<0.001
Valvuloplasty, *n* (%)	61 (41)	321 (56)	<0.001
Balloon dilation, *n* (%)	107 (72)	438 (77)	0.264
Cardiopulmonary resuscitation, *n* (%)	25 (17)	33 (5.8)	<0.001
Before rapid pacing, *n* (%)	1 (0.7)	7 (1.2)	1.000
After rapid pacing, *n* (%)	4 (2.7)	4 (0.7)	0.061
Before balloon dilation, *n* (%)	5 (3.4)	5 (0.8)	0.055
After balloon dilation, *n* (%)	1 (0.7)	1 (0.2)	0.370
Before valve deployment, *n* (%)	2 (1.4)	2 (0.4)	0.189
After valve deployment, *n* (%)	4 (2.7)	6 (1.1)	0.130
Defibrillation, *n* (%)	19 (13)	31 (5.4)	0.002
Heart–lung circulatory support, *n* (%)	9 (6.1)	14 (2.5)	0.035
Emergency Heart–lung circulatory support, *n* (%)	1 (0.7)	0 (0.0)	0.206
Conversion to open heart surgery, *n* (%)	3 (2.0)	11 (1.9)	1.000
Pericardial tamponade, *n* (%)	2 (1.4)	14 (2.5)	0.546
Valve malpositioning, *n* (%)	6 (4.1)	26 (4.6)	1.000
Coronary artery obstruction, *n* (%)	0 (0.0)	2 (0.4)	1.000
Ventricular septal perforation, *n* (%)	1 (0.7)	4 (0.7)	1.000

Data are expressed as mean ± standard deviation (SD), median (range) with interquartile range or percentage (count) as indicated.

**Table 3:** Postoperative characteristics.

Postoperative Characteristics	EF ≤ 40% (*n* = 148)	EF > 40% (*n* = 571)	*p*-Value
In-hospital mortality, *n* (%)	9 (6.1)	17 (3.0)	0.071
Cardiopulmonary	1 (0.7)	1 (0.2)	0.370
Major bleeding	3 (2.0)	2 (0.4)	0.062
Cerebrovascular	0 (0.0)	2 (0.4)	1.000
Sepsis, *n* (%)	6 (4.1)	14 (2.5)	0.272
Stroke, *n* (%)	5 (3.4)	10 (1.8)	0.208
Transient ischemic attack, *n* (%)	1 (0.7)	5 (0.9)	1.000
New onset of atrial fibrillation, *n* (%)	7 (4.7)	29 (5.1)	1.000
New atrioventricular block I°, *n* (%)	6 (4.1)	41 (7.2)	0.195
New atrioventricular block II°, *n* (%)	6 (4.1)	6 (1.1)	0.021
New atrioventricular block III°, *n* (%)	13 (8.8)	70 (12)	0.238
New pacemaker implantation, *n* (%)	17 (11)	67 (12)	0.933
In-hospital stay, days	10 ± 7.7	8.9 ± 5.4	0.402
Intensive care unit stay, days	4.4 ± 6.5	3.3 ± 3.5	0.298
Second intensive care unit stay, *n* (%)	13 (8.8)	39 (6.8)	0.414
Red blood cell transfusion, units	1.3 ± 2.8	0.8 ± 2.4	0.009
Paravalvular leakage, higher grade, *n* (%)	6 (4.1)	33 (5.8)	0.618
Aortic valve endocarditis, *n* (%)	3 (2.0)	2 (0.4)	0.062
Aortic valve thrombosis, *n* (%)	1 (0.7)	1 (0.2)	0.370
Left-ventricular ejection fraction, %	47 ± 12	60 ± 7.0	<0.001
Mean pressure gradient, mmHg	11 ± 5.1	12 ± 5.6	0.067
Peak pressure gradient, mmHg	21 ± 9.3	23 ± 9.7	0.101
Coronary artery obstruction, *n* (%)	0 (0.0)	2 (0.4)	1.000
Ventricular septal perforation, *n* (%)	1 (0.7)	4 (0.7)	1.000

Data are expressed as mean ± standard deviation (SD), median (range) or percentage (count) as indicated.

## Data Availability

Data are contained within the article.
